# „Kalkanogenese“ mit sekundärer Achillessehnen-Knochenblock-Alloplastik zur Wiedergewinnung der Rückfußfunktion: Eine 12-Jahres-Kasuistik

**DOI:** 10.1007/s00113-024-01458-6

**Published:** 2024-07-24

**Authors:** Hans Zwipp, René Grass, Michael Amlang, Stefan Rammelt

**Affiliations:** UniversitätsCentrum für Orthopädie, Unfall- und Plastische Chirurgie, Universitätsklinikum Carl Gustav Carus, Technische Universität Dresden, Fetscherstraße 74, 01307 Dresden, Deutschland

**Keywords:** 3° offene Kalkaneusluxationsfraktur (Typ 5), Subtotale Kalkanektomie, Taluscorpusdistraktionsosteotomie, Achillesehnentotalverlust, Homologer Achillessehnen-Knochenblock-Ersatz, 3rd degree open calcaneus fracture dislocation (type 5), Subtotal calcanectomy, Talar distraction osteotomy, Total Achilles tendon loss, Achilles tendon-bone block allograft

## Abstract

**Hintergrund:**

Kalkanektomie und Achillessehnenresektion sind kaum reparabel.

**Ziel der Arbeit:**

Zu zeigen, dass eine „Kalkanogenese“ mit OSG-Erhalt möglich ist und selbst nach 3,5 Jahren Functio laesa des M. triceps surae dieser nach homologem Achillessehnenersatz wieder aufbaubar ist.

**Material und Methode:**

Ein 25‑j. Motoradfahrer erlitt eine 3° offene Kalkaneusluxationsfraktur (Typ 5). Osteitis und Nekrose erforderten die Kalkanektomie, Resektion der Achillessehne und einen M.-latissimus-dorsi-Transfer. Eine Talusosteotomie mit Ilizarov-Distraktion schuf im 1. Schritt einen „Neokalkaneus“. In einem verzögerten 2. Schritt erfolgte zur Wiedergewinnung der aktiven Plantarflexion die Transplantation eines frisch gefrorenen Achillessehnen-Knochenblock-Allografts.

**Ergebnisse:**

Der AOFAS (American Orthopaedic Foot & Ankle Society)-Wert von initial 35 Punkten mit jetzt 70 Punkten 12 Jahre nach dem Ersteingriff spiegelt ein signifikant verbessertes Ergebnis. Mit beiden Maßnahmen gewann der Patient sein Gehvermögen ohne Orthese mit 88 % seiner normalen Plantarflexionskraft zurück. Die Angaben nach EQ-5D-5L (EuroQol-5 Dimension-5 Levels) zur Lebensqualität wurden auf der vertikalen EQ visuellen Analogskala vom Patienten mit 80 von 100 Punkten dokumentiert.

**Diskussion:**

„Kalkanogenese“ unter Erhalt des OSG (Oberes Sprunggelenk) ist möglich. Trotz langer Inaktivität des M. triceps surae von 3,5 Jahren kann ein homologer Achillessehnenersatz mit Knochenblock selbst an einem um ein Drittel kleineren Neokalkaneus 88 % der normalen Abstoßkraft wiederherstellen.

## Abstract (en)

**Background:**

Calcanectomy and Achilles tendon resection are very hard to repair.

**Objective:**

Ilizarov’s “calcaneogenesis” is possible with ankle joint preservation. Even after 3.5 years of functio laesa of the triceps surae muscle it can be rebuilt.

**Material and methods:**

A 25-year-old motorcyclist suffered a 3rd degree open calcaneal dislocation fracture (type 5). Osteitis and necrosis required calcanectomy, resection of the Achilles tendon and a latissimus dorsi muscle transfer. A talus corpus osteotomy with Ilizarov distraction created in the 1st step a “neo-calcaneus”. In a delayed 2nd step a fresh-frozen Achilles tendon-bone block allograft was transplanted to regain active plantar flexion.

**Results:**

The initial AOFAS score of 35 points was significantly improved to 70 points 12 years after step 1. After both operations the patient could walk without an orthosis and regained 88% of normal plantar flexion strength. Quantitative measure of health outcome according to EQ-5D-5L was marked by the patient with 80 out of 100 points.

**Discussion:**

“Calcaneogenesis” with preservation of the ankle joint is possible and innovative. Despite 3.5 years of disconnection of the triceps surae muscle, an Achilles tendon-bone block allograft could restore 88% of the push-off force even attached to a neo-calcaneus that is 1/3 smaller than normal, which is also new.

Die Evolution brauchte Millionen Jahre zur Entwicklung des menschlichen Fußes als Prärequisit für den aufrechten Gang. Das humane Fersenbein als größter tarsaler Knochen nimmt rückseitig die kräftigste Sehne des menschlichen Körpers auf, wodurch beide in ihrer Interaktion Garant fürs freie Gehen und seit der Antike Symbol des Lebens in Freiheit sind. Dieses geniale Geschenk der Natur kann innerhalb von Millisekunden dem Individuum verlorengehen, wenn durch einen Unfall beide Elemente des Rückfußhebels wie im vorliegenden Fall aufgrund einer foudroyanten Infektion durch radikales Débridement zum Lebens- und Extremitätenerhalt verlorengehen.

## Fallvorstellung

### Anamnese, Diagnosen und Befund

**Abb. 1:**
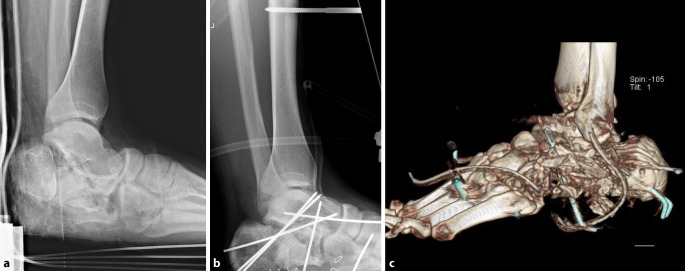
Radiologisch serielle Frakturen des linken Rück- und Mittfußes. **a** Kalkaneusluxationsfraktur (Typ 5, 3.° offen) mit Kuboidimpressionsfraktur und Lisfranc-Brüchen. Aufnahme am Tag des Unfalls. **b** Notversorgung mit Débridement, Adaptations-K-Draht-Osteosynthese, tibiotarsaler Transfixation und Kunsthautdeckung. **c** CT-Kontrolle. (2 Tage nach Unfall)

Ein 25-jähriger Motorradfahrer erlitt bei einem Hochenergietrauma durch Kollision mit einem Traktor ein schwerstes Barytrauma des linken Fußes. Nach Notversorgung am Unfallort erfolgte die Primärversorgung an der regionalen Universitätsklinik. Dort wurden eine drittgradig offene Kalkaneusluxationsfraktur (Typ 5) [1] links radiologisch mit ipsilateraler Kuboidimpressionsfraktur und multiplen Lisfranc-Brüchen festgestellt (Abb. 1a), als Nebenbefund eine Klavikula- und Mittelhandfraktur links.

Ein anfänglicher Erhaltungsversuch des linken Rückfußes mit Débridement, adaptiver K‑Draht-Osteosynthese, entlastender tibiotarsaler Transfixation des Fußes und Kunsthautdeckung (Abb. 1b, c) scheiterte aufgrund einer foudroyant einsetzenden Mischinfektion des Fersenbeines mit umgebenden Weichteilen, was radikale Débridements mit Kalkanektomie, Achillessehnenresektion und anderer Weichteilstrukturen erforderte. Als 5. Operation wurde ein freier M.-latissimus-dorsi-Lappen mit teils großen Mesh-Graft-Arealen zum Extremitätenerhalt durchgeführt.

### Erstvorstellung 6 Monate nach dem Unfall

**Abb. 2:**
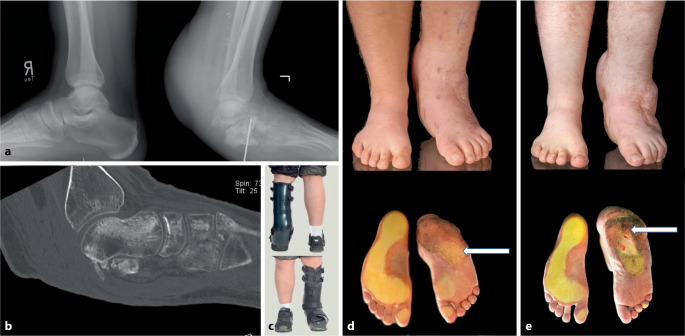
**a**–**d** Erstvorstellung 6 Monate nach dem Unfall mit 5 Voroperationen, zuletzt mit M.-latissimus-dorsi-Lappen **a**, **b** Seitliche Belastungsaufnahmen zeigen die subtotale Kalkanektomie, eine Tibiainklination mit verbliebenem K‑Draht. Das CT (**b**) zeigt eine fragliche avaskuläre Nekrose bzw. schwere Demineralisierung des Talus, **c** Spezialorthese, **d** Spiegeltisch-Aufnahme (präoperativ) mit fehlender knöcherner Ferse, Mittfußlastaufnahme (*Pfeil*), **e** 8 Monate nach Kalkanogenese mit erkennbarer Rückfußvergrößerung und Dorsalisierung der Lastaufnahme (*Pfeil*)

Bildgebend bestanden nur Reste des Processus anterior calcanei lateralseitig und des gelenknahen Sustentaculum tali medialseitig (Abb. 2a, b). Gehfähigkeit bestand nur mit einer speziellen Orthese (Abb. 2c). Die Spiegeltischaufnahme (Abb. 2d) zeigte das Fehlen der knöchernen Ferse, die pathologische Lastaufnahme im Mittfußbereich und die mächtige Lappenauftreibung distal mit völlig atrophierter Wade links.

### Therapieoptionen

Zur rein konservativen Orthesenbehandlung, zur Endo-Exo-Prothetik wurde dem Patienten als dritte Alternative die Ilizarov-Kalkanogenese unter Erhalt des OSG angeboten. Diese erfahrungsgemäß langwierige, die Mitarbeit des Patienten erfordernde, mit etlichen stationären Aufenthalten verbundene und zum Heilergebnis ungewisse Talusosteotomie wünschte der Patient dezidiert, trotz des Aufwands und aller Risiken.

## Therapie und Verlauf

Nach Entfernung eines verbliebenen K‑Drahts (Abb. 2a) mit jetzt möglicher Angio-MRT des Fußes und distalen Unterschenkels links am Heimatort konnte eine avaskuläre Nekrose des Talus ausgeschlossen, es konnten aber auch weder Reste der Achillessehne noch der Flexor-hallucis-longus-Sehne nachgewiesen werden.

### Kalkanogenese

**Abb. 3:**
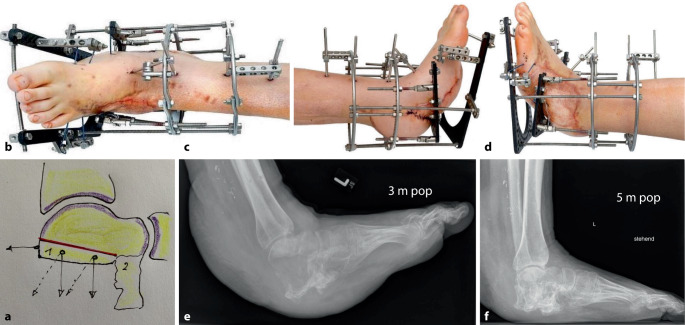
**a** Talusosteomie-Planung 9 Monate nach dem Unfall: *1* Keil dorsal 1,5, ventral 1 cm, *2* Rest des Processus anterior calcanei. **b**–**d** Ilizarov-Montagen. Osteotomie des Talus **e** Sichtbarer Kallus nach Entfernung des Fixateurs 3 Monate postoperativ und Verordnung von gepulstem Ultraschall. **f** Kallusdurchbau nach 5 Monaten postoperativ unter zunehmender Vollbelastung im halbhohen Sportschuh

Neun Monate nach dem Unfall wurde in Bauchlage dorsozentral zum Talus zugegangen, der Taluskörper wurde dorsal wie geplant (Abb. 3a) 1,5 cm oberhalb der posterioren Facette mit breitem, scharfen Meißel osteotomiert. Der nach distal sich verschmälernde Knochenkeil wurde mit 3 Olivendrähten gefasst, die Ilizarov-Montage unter sterilen Kautelen anschließend in Rückenlage vervollständigt (Abb. 3b–d). Nach 5 Tagen wurde der tägliche 1‑mm-Transfer begonnen, in dessen Handhabung, inklusive der Pin-Pflege, der Patient und dessen Vater detailliert eingewiesen wurden. Trotz penibler Pin-Pflege durch den Patienten musste nach 3 Monaten am Heimatort bei Pin-Infektionen die Entfernung des Fixateurs erfolgen, mit sich jetzt anschließender gepulster Ultraschallbehandlung (Abb. 3e). Nach 2 weiteren Monaten war unter zunehmender Vollbelastung im halbhohen Sportschuh der Kallus fest durchbaut (Abb. 3f).

### Interimsphase mit Abwägung weiterführender Therapieoptionen

Da der Patient 6 Monate nach der *Kalkanogenese* in halbhohen Sportschuhen 3 km schmerzfrei gehen konnte, er als Maschinenbau-Ingenieur seit 2013 voll arbeitsfähig war, ein M.-flexor-hallucis-longus-Transfer bei Verlust und auch eine M.-peronaeus-brevis-Transposition zur Wiedergewinnung der Abstoßkraft bei dorsal zu kurzem „Neokalkaneus“ biomechanisch nicht sinnvoll erschienen, blieb als letzte Option nur die eines homologen Achillessehnen-Knochenblock-Transplantats. Da aber die schwere Rückfußinfektion erst eineinhalb Jahre zurücklag, die Gesamtsituation des Patienten bei komplikationsloser *Kalkanogenese* mit Wiedergewinnung der Gehfähigkeit im halbhohen Sportschuh von 3 km möglich geworden war, eine Verfünffachung der Abstoßkraft nachgewiesen wurde, eine Erholung des fettig degenerierten M. triceps surae nach 18 Monaten Inaktivität wenig wahrscheinlich erschien und die einzig verbleibende Therapieoption eines homologen Sehnen-Knochen-Transplantats ein Reinfektionsrisiko darstellte, rieten wir dem Patienten zunächst zur weiteren konservativen Behandlung, um bisher Gewonnenes nicht zu verlieren.

### Frisch gefrorene, homologe Achillessehnen-Knochenblock-Transplantation

**Abb. 4:**
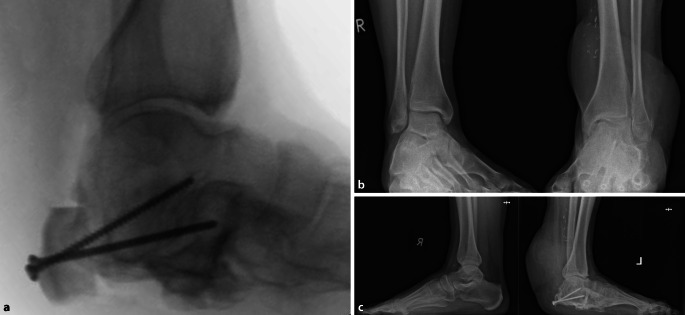
Minimal-invasive Achilles-tendon-bone-allograft-Transplantation 3,5 Jahre nach Unfall (**a**). Röntgenbelastungsaufnahmen im Stehen a.-p. (**b**) und seitlich (**c**) 12 Jahre nach Talusosteotomie und knapp 10 Jahre nach homologer Achillessehnen-Knochenblock-Transplantation (**c**). Mäßige OSG-Arthrose links

Da der junge Patient jedoch wiederholt auf Verbesserung seiner persönlichen Bewegungsfreiheit mit sportlichen Optionen wie Fahrradfahren drängte, alle Risiken und selbst den ungewissen Erfolg einer homologen Transplantation für sich in Kauf nehmen wollte, führten wir 3,5 Jahre nach seinem Unfall diesen Eingriff minimal-invasiv durch (Abb. 4a). Das am Tag der Operation frisch gefrorene Achillessehnenpräparat mit Knochenblock (Größe 250/225/15) vom Deutschen Institut für Zell und Gewebeersatz (DIZG), Berlin, wurde aufgetaut.

In Bauchlage wurde unter sterilen Kautelen medialseitig unter Schonung der medialen Lappengefäße der große Sehnenspiegel nach Tunnelierung mit stumpfer Kornzange von distal nach proximal positioniert, dort verkleinert und später nach Platzierung und Verschraubung des Knochenblocks (zwei 3,5-mm-Kortikalisschrauben) bei 30°-Spitzfußstellung auf den originären Triceps-surae-Spiegel mit resorbierbarer Naht aufgesteppt. Der 5 × 4 × 1 cm große homologe Fersenblock musste medial in situ noch verkleinert und abgerundet werden, damit sich der lokal mobilisierte und mit Mesh-Graft-gedeckte M.-latissimus-dorsi-Lappen gerade noch darüber zunähen ließ.

## Ergebnis

### Klinisch

ist das Gangbild bei der 12-Jahres-Kontrolle in flachen Sportschuhen normal und flüssig. Der Einbein-Zehenspitzen-Stand ist links gegenüber rechts eingeschränkt, aber ausführbar. Die Großzehe links kann nicht eingekrallt werden, die Kleinzehen beugen mittelgradig. Die Ersatzfußsohle fühlt sich für den Patienten fest und trocken an. Mit flachen Turnschuhen und dämpfender Sohle werden eine Gehstrecke von mehr als 3 km und unbegrenztes Radfahren angegeben. Der AOFAS von initial 35 mit jetzt 70 Punkten spiegelt ein signifikant verbessertes Ergebnis. Der FFI-Test zeigt auf der Funktionsskala von 0–9 einen Mittelwert von 4,5. Die Angaben nach EQ-5D-5L zur Lebensqualität werden auf der vertikalen EQ visuellen Analogskala vom Patienten mit 80 von 100 Punkten dokumentiert.

### Radiologisch

**Abb. 5:**
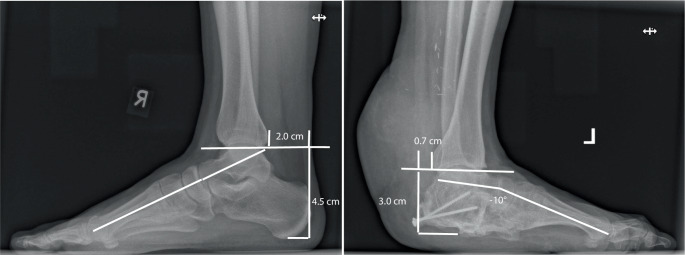
Seitliche Röntgenbelastungstaufnamen wie Abb. 4c mit erkennbarer Reduktion des Knochenblocks. Im Seitenvergleich ist die Rückfußhöhe bei gleichem Maßstab links von 3,0 cm gegenüber 4,5 cm rechts um ein Drittel gemindert, der Rückfußhebelarm links mit 0,7 cm gegenüber 2,0 cm rechts um annähernd ein Drittel verkürzt, die talometatarsale Achse ist links bei unzureichender Höhe des Neokalkaneus um 10° inkliniert

hat der heute 37-jährige Patient 12 Jahre nach der *Kalkanogenese* einen um ein Drittel minder hohen *Neokalkaneus* links zurückgewonnen (Abb. 4 und 5) sowie knapp 10 Jahre nach homologem Achillessehen-Knochenblock-Ersatz einen Rückfußhebel, der allerdings um ein Drittel kürzer und um ein Drittel niedriger ist als der gesunde rechte (Röntgenbildmessung 2,0 cm vs. 0,7 cm bzw. 4,5 c vs. 3,0 cm; Abb. 5). Links besteht eine mäßige Arthrose des OSG bei minderhohem *Neokalkaneus* (Abb. 4b, c und 5).

### Apparativ

**Abb. 6:**
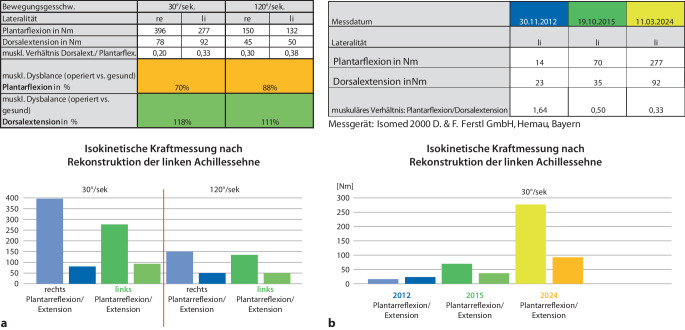
**a** Isokinetische Kraftmessung im Seitenvergleich 12 Jahre postoperativ. Trotz des verkürzten Rückfußes erreicht der linke rekonstruierte Rückfuß für die aktive Plantarflexionskraft bei 30°/s immerhin 70 %, bei 120°/s sogar 88 % der gesunden Seite. Die Dorsalextensionskraft ist wegen des verkürzten Hebelarms sogar größer als rechts. **b** Die Kraftmessung im Verlauf (2012, 2015, 2024) zeigt nach der Kalkanogenese eine Verfünffachung der Plantarflexionskraft, nach dem Achillessehnenersatz eine Verzwanzigfachung. Patientendaten zur Zeit der Messung bei der 12-Jahre-Kontrolle 2024: Alter 37 Jahre, Größe 179 cm, Gewicht 108,5 kg, BMI 33,9, Fett 29 %

zeigt die Kraftmessung für die Plantarflexion im Isomed-2000-Messgerät (Abb. 6) im Verlauf von 2012–2024 eine Verfünffachung der Abstoßkraft (14 vs. 70 Nm) allein durch die *Kalkanogenese*. Durch die zusätzliche Transplantation des homologen Achillessehen-Knochenblocks wurde eine Verzwanzigfachung (14 vs. 277 Nm, f = 19,79) erzielt.

### Psychosozial

ist der Patient seit 2013 als Ingenieur dauerhaft voll arbeitsfähig. Er ist inzwischen verheiratet und Vater von 4 Kindern. Für ihn habe sich der operative Aufwand sehr gelohnt; eine Amputation habe er nie gewollt. Er ist mit seinem gesundheitlichen Zustand sehr zufrieden. Die ärztliche Empfehlung zur Gewichtsreduktion zwecks Entlastung des OSG links wird angenommen.

## Diskussion

In der Literatur sind nur wenige Berichte einer *Kalkanogenese* [2–5] bekannt, wobei unter Osteotomie der dorsalen gelenkbildenden Tibia in Kombination mit einer Talusosteotomie [2, 4, 5] eine zwangsläufig resultierende OSG-Arthrodese in Kauf genommen wird. Dies sollte im eigenen Vorgehen aber *de principe* vermieden werden. Retrospektiv hätte eine mehr keilförmige Osteotomie des Taluscorpus (dorsal 2,5 cm statt 1,5 cm) mit stärkerer Oliven-K-Draht-Zugrichtung nach dorsoplantar (Abb. 3a) statt nur nach plantar zu einem größeren Hebelarm des Neokalkaneus führen können, was aber zum sicheren Erhalt des knorpeligen dorsalen OSG-Abschnitts bewusst vermieden wurde. Ein vaskularisierter Knochenaufbau des Fersenbeins nach der Kalkanektomie mit gleichzeitiger Weichteildeckung mittels M.-serratus-anterior-Transfer, wie früher beschrieben [6], erschien alternativ bei bereits eingeheiltem M.-latissimus-dorsi-Transfer nicht opportun.

Boorboor et al. [7] konnten nach Resektion infizierter Achillessehnen in 7 Fällen zeigen, dass nach plastischer Deckung ohne Ersatz der Achillessehne nach fast einem Jahr die plantare Kraftmessung bei 120°/s i. M. um 46 % vermindert war. Heikkinen et al. [8] konnten 14 Jahre nach der Achillessehnennaht nach akuter Ruptur bei MRT-Messungen sogar feststellen, dass Volumenmessungen des M. soleus, des medialen und lateralen M. gastrocnemius um 63 cm^3^ hochsignifikant (< 0,001) geringer waren als die auf der nichtoperierten Seite. Die gemessene Plantarflexionskraft war um 12–18 % gegenüber der gesunden Seite im Mittel gemindert.

2014, zur Zeit der allogenen Achillessehnen-Knochenblock-Transplantation im vorgestellten Fall, lagen noch keine Zahlen zum Infektionsrisiko solcher Allografts vor. Spätere Berichte [9, 10] zeigen beispielhaft, dass bei anderen Konditionen wie bei Ersatzoperationen aufgrund der Resektion bei chronischer Ruptur mit großen Defekten, wegen lokal-entzündlicher oder tumoröser Prozesse, keine postoperativen Komplikationen i. S. einer Infektion beobachtet werden.

## Fazit für die Praxis

Die Wiederherstellung des seltenen Komplettverlusts von Fersenbein und Achillessehne als Prärequisit fürs freie Gehen ist an speziellen Zentren operativ möglich, erfordert aber große Compliance und Mitarbeit des Patienten.
